# Supplementary material for: Sustained Complete Response after Biological Downstaging in Patients with Hepatocellular Carcinoma: XXL-Like Prioritization for Liver Transplantation or “Wait and See” Strategy?
Source: Cancers (Basel). 2021 May 17;13(10):2406. doi: 10.3390/cancers13102406 (PMC8156031; doi:10.3390/cancers13102406)
Supplement: Supplementary file 1 [file cancers-13-02406-s001.zip › cancers-1144002-SI.pdf]

cancers

MDPI

XXL protocol inclusion and exclusion criteria (https://ars.els-cdn.com/content/image/1-s2.0-S1470204520302242-mmc1.pdf)

Inclusion Criteria

• Cirrhotic patients (any etiology), age 18-69 years, within Child-Pugh class: A-B7

• ECOG Performance Status 0-1

• Confirmed diagnosis of HCC according to EASL-AASLD criteria

• HCC exceeding Milan Criteria with a 5-yr estimated survival >50% after transplantation according to the Metroticket calculator (www.hcc-olt-metroticket.org).

• Previous diagnosis and treatments of HCC:

- First diagnosis of previously untreated HCC maximum 6 months prior to first downstaging treatment.
- Patients who initiated treatments for HCC (complying with the aforementioned criteria) ≤18 months before enrolment, either at the recruiting Center or elsewhere.

• Recurrent HCC after curative treatments (ie, only after surgical resection and radiofrequency ablation):

- New occurrence of HCC, providing the radiological demonstration of complete tumor response after the previous curative treatment
- Late recurrent HCC, (ie, patients with at least two years span from the end of previous curative treatments) providing demonstration that the recurrent intrahepatic tumor exceeds Milan Criteria and has a 5-yr estimated survival >50% at the Metroticket calculator
- Early recurrent HCC, (ie, less than two years span from the end of previous curative treatments), particularly in case of:
  - HCC within Milan at the time of first treatment, while exceeding Milan at the cumulative tumor staging (i.e. the HCC stage resulting from the sum of first occurring + recurrent HCC)
  - Survival prediction above 50% at 5 yrs, when loading in the Metroticket calculator the cumulative tumor characteristics

• Signed informed consent for the study.

• Women of child bearing potential with a negative serum pregnancy test performed before enrolment

• Absence of general contraindications to sorafenib treatment

Exclusion Criteria

• Presence of extra-hepatic spread (EHS) defined as organ involvement other than the liver. That is:

- Hepatic hilum lymph-nodes with short axis > 2 cm
- Presence of macrovascular invasion defined as:
  - PVT with invasion of main trunk, or left/right branches (type 2-4 according to Shi 2010),
  - concomitant AFP level at any value above 400 ng/mL
  - Invasion of vena cava or main trunks of hepatic veins

• Patients who have already achieved partial or complete response after downstaging ended more than 3 mo before enrolment

• Patients in sorafenib treatment, if started and maintained for > 2 months before enrolment

• Previous or concurrent cancer that is distinct in primary site or histology from HCC, except cervical carcinoma in situ, treated basal cell carcinoma, superficial bladder tumors (Ta, Tis, T1).

• Other cancers curatively treated < 5 years from study entry

• Active intra-venous or alcohol abusers (patients may be eligible if abstention > 6 months is demonstrated)

• HIV infection; HBV-DNA > 20.000 UI/mL; active clinically serious infections, except for HCV and HBV

• History of cardiac disease:

- Congestive heart failure > New York Heart Association (NYHA) Class II

- Active coronary artery disease (CAD) (myocardial infarction >6 mo. prior to study entry is allowed)
- Cardiac arrhythmias (> Grade 2 NCI-CTCAE Version 4.0) which are poorly controlled with antiarrhythmic therapy or requiring pace-maker

• Severe pulmonary hypertension, with PAM ≥ 45mmHg, not treatable with medical therapy – Hepatopulmonary disease with SO_2<50%

• Psychiatric disorders, if not adequately supported by medical treatment and family.

• Severe neurological diseases (Alzheimer disease etc.)

• Patients with a life expectancy of less than 3 months due to HCC or less than 6 months due to any other disease
